# Work Interruption Experienced by Nurses during Medication Administration Process and Associated Factors, Northwest Ethiopia

**DOI:** 10.1155/2017/8937490

**Published:** 2017-11-20

**Authors:** Mehammed Adem Getnet, Berhanu Boru Bifftu

**Affiliations:** Department of Nursing, University of Gondar College of Medicine and Health Science, Gondar, Ethiopia

## Abstract

**Background:**

During medication administration process, including preparation, administration, and documentation, there is high proportion of work interruption that results in medication administration errors that consequently affect the safety of patients. Thus, the main purpose of this study was to assess the prevalence of work interruption and associated factors during medication administration process.

**Methods:**

A prospective, observation-based, cross-sectional study was conducted on 278 nurses. Structure observational sheet was utilized to collect data. EPI Info version 3.5.3 and SPSS version 20 software were utilized for data entry and analysis, respectively. Binary and multivariable logistic regression were fitted to identify the associated factors using an odds ratio and 95% CI.

**Results:**

The incidence of work interruption was found to be 1,152 during medication administration process. Of this, 579 (50.3%) were major/severe work interruptions. Unit of work, day of the week, professional experience, perceived severity of work interruption, source/initiator of interruption, and secondary tasks were factors significantly associated with major work interruptions at *p* < 0.05.

**Conclusion:**

In this study, more than half of work interruption was major/severe. Thus, the authors suggest raising the awareness of nurses regarding the severity of work interruptions, with special attention to those who have lower work experience, sources of interruption, and secondary tasks by assigning additional nurses who manage secondary tasks and supportive supervision.

## 1. Background

Patient safety is a key aspect in the care of the patients and one of the dimensions to determine the quality of care [1, 2]. Medication errors have been identified as one of the most common type of errors affecting patient safety, ranging from 42 to 59% of all medical errors [3]. The nursing medication administration process involves the preparation, administration, and documentation in a continuous process through a series of actions without interruption [4–7]. However, the medication administration process remains the most interrupted nursing activity globally [8, 9]. Work interruptions (WIs) are a break in the continuity of task performance [10] and result in the task being unexpectedly suspended at some step prior to completion [11] due to distraction or intrusion of unplanned secondary tasks or demands [7, 12, 13]. Studies revealed that work interruptions during medication administration rounds are thought to be a prominent factor in medication errors that account for up to 43% of medication errors [14]. Studies showed that nurses are interrupted at a rate of 5.5 to 14 work interruptions/hr [15, 16], these causing a high level of inefficiency as nursing was not meeting its goal of delivering all medication within 60 minutes of prescribed acceptable time and creating a significant risk that distracted/interrupted nurses leading to them making mistakes or errors [17]. Work interruptions during the medication administration process usually lead to clinical errors in nursing practice with up to 88.9% having negative consequences [18]. Work interruptions also associated with a 12.1% increase in procedural failures and a 12.7% increase in clinical errors [19] and contributed to an average of 2.1-hour time loss per day, and the subsequent lost productivity cost to economy was $588 billion dollars per year from the general working environment [20, 21]. Beyond its effects on patient safety and resources, work interruptions also affect the general well-being of employees [22], for example, increased anxiety, stress, and dissatisfaction, and contribute to high workload [11, 22]. Evidence showed that work interruptions result from self-initiation (the individual nurse him/herself on the medication administration process task), other persons' initiation, system failure (missing medications, missing equipment), environmental noise, and occurrence of emergency situation in the nursing environment; work experience was associated with work interruptions [23].

In Ethiopia, where there is lack of educated healthcare professionals, low nurse-to-patient ratio, lack of different material and financial resources, and high patient flow, evidences indicated that the incidence of medication error is a common problem that ranges from 4.35 to 89.9% [24, 25]. There is also an evidence that revealed the relation between work interruption and medication administration errors [26]; in most of the medication administration errors, appropriate measures, such as error reporting, were not taken [27]. Evidences on the magnitude and contributing factors have significant impact on the design of corrective intervention that helps to maximize medication safety within organizations [14, 28, 29]. Despite the impact of work interruptions and its high magnitude, there is no well-documented study in Ethiopia. Therefore, the aim of this study was to assess the proportion of work interruption and associated factors during medication administration process in Northwest Ethiopia.

## 2. Methods

### 2.1. Study Design and Period

Cross-sectional study design was used from April 7 to May 7, 2017.

### 2.2. Study Setting

The study was undertaken in Amhara Regional State, Northwest Ethiopian. In Amhara Regional State, there are three referral hospitals: Debre Markos Referral Hospital (DMRH), Felege Hiwot Referral Hospital (FHRH), and Gondar University Referral Hospital (GURH).

### 2.3. Source and Study Population

All nurses working in the referral hospitals of the Amhara Regional State were the source of the study, and those nurses included in the study were the study population. All staff nurses who have work experience of greater than or equal to six months were included in the study.

### 2.4. Sample Size Determination and Sampling Procedure

The sample size of the study was determined using the following formula: *n* = *z*^2^*p*(1 − *p*)/*W*^2^, where *P* = 0.5, *W* = 0.05, and *Z* = 1.96 (i.e., for a 95% CI), and since the sample above was taken from population (*N* = 949), the required minimum sample size was adjusted using correction formula and, considering 10% nonresponse rate, the final sample size became 301. For sampling, first proportional allocation was carried out for the three hospitals and for the five different units of the hospitals. Nurses were eventually distributed through 7-day week (Monday–Sunday) and eight different time schedules of medication round such as 12 PM, 2 PM, 6 PM, 10 PM, 12 AM, 2 AM, 6 AM, and 10 AM. Therefore, finally, the sampled nurses were selected randomly by lottery methods during their medication round events on both night and day work shifts.

### 2.5. Data Collection Instruments and Procedure

Six data collectors and one supervisor were trained for one day, two weeks before the* beginning* of actual data collection date, on the objectives of the study, the format of the questionnaire and checklist, procedures of observation, and methods of reporting to supervisors and principal investigators. Two observers were collecting data from a single participant to minimize observation bias, and finally they reach consensus through discussion if disparity was observed. A structured observation sheet was used for collecting the frequency and sources of work interruptions using observational method and face-to-face interview method for the sociodemographic variables and perceived severity of work interruption. The structured observation sheet/checklist and the assessment tools of sociodemographic and perceived severity of work interruption were developed by the authors through reviewing similar studies [9, 15, 22, 23, 30–33]. The appropriateness of the instruments was measured through pretesting and inviting different experts in the field of nursing to review or evaluate the instruments. A chronometer (stop watch) was also used to monitor and record the duration of work interruptions. The content resulting from data collection was designed to record the nurses' sociodemographics, work experience, data on environmental variables, and date and time when work interruptions were observed including the time schedules of the medication round such as 12 PM, 2 PM, 6 PM, 10 PM, 12 AM, 2 AM, 6 AM, and 10 AM, in addition to reasons for and sources of work interruptions. The medication round was considered to begin when the nurse opened the medication trolley and end when all medications were administered.

In this study, work interruption was defined as a break in the continuity of task performance (i.e., medication administration process) and results in the task being unexpectedly suspended and/or stopped at some step prior to completion [11], and it is classified as major or minor based on (a) the time/duration and (b) the effect of work interruptions. The time bound/duration is the time elapsing between the start and the end of work interruption or resuming the activity previously interrupted/distracted (i.e., medication administration process), and it is classified as (i) prolonged interruption (interruption of the medication administration process activity that lasted for 1–5 minutes, but the nurse participant resumes the medication administration process activity after the end of that interruption), (ii) extensively prolonged interruption (interruption of the medication administration process activity that lasted for >5 minutes, but the nurse participant could still resume the medication administration process activity after the end of that interruption), and (iii) abandoned activity (the nurse participants could not resume the specific one medication administration process activity after interruption). Thus, major work interruption was defined as work interruption that lasted for ≥1 minute, including prolonged interruption, extensively prolonged interruption, and abandoned activity, while minor work interruption was defined as work interruption that lasted <1 minute, including distraction only, momentary pause, and nonprolonged interruption. “Distractions only” means that the distraction occurred during the nurses' medication administration process activity/task, but the nurse participant could perform the medication administration process without any pause (no observable effect/pause on the medication administration process). “Momentary pause” means that the participating nurse resumed the activity after momentary pause that lasted for ≤10 seconds and then either the distraction ceased or the medication administration process was resumed during that distraction and interruption. “Nonprolonged interruption” means that the interruption of the medication administration process activity lasted for 11–59 seconds, but the participating nurse resumed the activity after the end of that interruption. Data was collected through observation of nurses carrying out medication administration.

### 2.6. Data Processing and Analysis

Data cleanup and crosscheck were carried out before the analysis. EPI Info version 3.5.3 statistical software and SPSS version 20 program were used for data entry and analysis, respectively. Descriptive (frequency, percent, mean, standard deviation, and tables) and analytic statistics were employed for data analysis and presentation. Bivariate and multivariate logistic regressions were used to identify the factors associated with work interruptions using an adjusted odds ratio with 95% confidence interval. All factors with a *p* value < 0.2 in the bivariate logistic regression were entered into the multivariate model to control confounders. From multivariate logistic regression, variables with *p* value < 0.05 were accepted.

### 2.7. Ethical Consideration

The study was conducted after receiving ethical approval from the Ethics Committee of the Department of Nursing at University of Gondar. In addition, letters of approval were obtained from Amhara Regional State head of research and technology transfer and submitted to the respective hospital. Verbal and written consent were obtained from participants.

## 3. Results

### 3.1. Sociodemographic Characteristics of the Study Subjects

A total of 278 nurses participated in this study with response rate of 92.4%. Out of 278 study subjects, 140 (50.4%) were men, the majority (190; 68.3%) were within the age range of 26–30, and 166 (59.7%) of participants were single. Regarding the educational status of participants, the majority (271; 78.1%) had B.S. degrees, and 163 (58.6%) had work experience of ≤5 years (Table 1).

### 3.2. Work Interruption

The incidence of work interruption was found to be 1,152 during the MAP: distraction only, 102 (8.9%); momentary pause, 156 (13.5%); nonprolonged interruption, 315 (27.3%); prolonged interruption 471 (40.9%); extensively prolonged interruption, 86 (7.5%); abandoned activity, 22 (1.9%).  Table 2 shows the details of work interruption during preadministration, administration, and postadministration phases. Overall, 579 (50.3%) cases of work interruption were major/severe. Of 278 study participants, the majority (222; 80%) experienced work interruptions more than two times.

Regarding the incidence of work interruption in working units, the highest incidence was found in the pediatric unit (Table 3).

### 3.3. Factors Associated with Major Work Interruptions

From the bivariate logistic regression analysis, the factors found to be associated with major work interruptions were type of inpatient unit, day of week, source of distraction/interruption, secondary task, age of the participant, professional experience of the participant, and participant's perception of the risks associated with major work interruptions. The multivariate analysis showed that in the weekends (Saturday, Sunday) nurses were nearly 1.4 times more likely to experience major work interruptions compared to weekdays (Monday–Friday); *p* < 0.05 (Table 4).

## 4. Discussion

Work interruptions are common and frequently cause public problem during the nursing medication administration process [9, 23, 34], usually having a negative consequences on patients' safety and outcome, employees' well-being and performance, and country's resources as a whole [19–22, 35]. Thus, the main aim of this study was to assess the incidence of work interruption and associated factors in Amhara Regional State. Overall, 1152 work interruptions were found within 286 hours and 12 minutes of direct observation. Of 1152 work interruptions, 102 (8.9%) were distraction only, 156 (13.5%) were momentary pause, 315 (27.3%) were nonprolonged, 471 (40.9%) were prolonged, 86 (7.5%) were extensively prolonged, and 22 (1.9%) were abandoned activity. Although directly comparing our results with those of other studies is difficult due to the variations in definitions, instruments, data collection methods, working environments, and participants' characteristics, the results of the present study were similar to those of another one [36], in which 1170 work interruptions were found during 300 hours of observation. Thus, this result supports the incidence of work interruption revealed by other studies [33].

Regarding the severity of work interruptions, 50.3% of the study participants experienced major/severe work interruptions. This finding was consistent with those of prior studies in Australia (53.1%) [19] and Canada (59.3% intrusions, 28.4% distractions) [18]. On the other hand, work interruption incidence reported in this study was higher than that shown by a study carried out in Italy (32%) [23]. This variation may be due to the variations in definitions, instruments, data collection methods, working environments, and participants' characteristics.

Regarding the factors associated with work interruptions during the nurses' medication administration process, unit type, day of week, source/initiator, secondary task/reason, nurses' professional experience, and nurses' risk perception of work interruptions were found to be significantly related to major work interruptions during the medication administration process.

Those nurses who work on weekend were around two times [AOR = 1.389, 95% CI: 1.021–1.890] more likely to experience major work interruptions when compared to those working on weekdays. This result is supported by other studies [37]. This may be due to the fact that social relationships especially in our context develop during holydays including weekends. The other possible explanation may be that the number of assigned nurses in the weekend was small; this may lead them to take on other secondary tasks usually performed by other nurses during the regular working schedules of weekdays. This explanation was also supported by our findings, as the most frequent sources of or reasons for interruption were face-to-face conversations, 66.6%; technical problems, 24.9%; and phone call, 8.5%. These reasons were also supported by the results of the analysis, as those nurses agreeing with phone calls were two times and those agreeing with technical problems were 1.5 times more likely to experience work interruptions compared to those who disagreed. These results were also supported by other studies [9, 14, 18, 32, 38]. Concerning technical problems faced by nurses during the medication administration process, a prior observational descriptive study reported that problems in the use of objects, failure of equipment, and missing medications and equipment impact workflow [39]. Another study also indicated that technical problem is also one of the most frequent and prolonged sources of interruptions during the nurses' medication administration process [32].

Regarding secondary tasks, nurses undertaking clerical activity were four times more likely to experience major interruption compared with those undertaking direct patient care activity (AOR = 4.250, 95% CI: 2.277–7.933). This is supported by another study [37].

Nurse participants who had professional experience of ≥5 years and 6–10 years were 2 times (AOR = 2.088, 95% CI: 1.277–3.414) and more than one time (AOR = 1.821, 95% CI: 1.078–3.075), respectively, more likely to experience major work interruptions compared to those who had greater than 10 years of professional experience. This could imply that nurses having less professional experience may be less capable of managing interrupting factors faced during their medication administration process; this leads to long interruptions. This is supported by other studies [37, 40, 41].

Those nurses who perceived the risk of work interruptions as low were around two times (AOR = 1.979, 95% CI: 1.504–2.605) more likely to experience major work interruptions when compared to those who perceived work interruptions as a high risk causing medication errors. A prior study also reported that most of professionals do not often perceive interruptions to be negative, except when they are unnecessary or disturb the work processes [42]; this may contribute to major work interruptions during the medication administration process.

## 5. Strength and Limitations of the Study

This is the first, to the best of the authors' knowledge, method used to collect data, and the techniques used to minimize observational bias and inclusion of data from the preadministration, administration, and postadministration phases of medication administration are considered as the strongest part of the study. On the other hand, since participants were informed of the objectives and intervened when interruptions were observed, the true proportion of work interruptions that nurses may experience may be minimized.

## 6. Conclusion and Recommendations

In this study, more than half of work interruptions were major/severe. Unit of work, day of week, source of distraction/interruption, secondary tasks, professional experience of the participants, and participants' perception of work interruption risks were associated with major work interruptions. Thus, the authors suggest raising the awareness of the consequences of work interruptions, with special attention to those who have lower work experience, and design strategies to manage secondary tasks such as direct care; teaching/supervising students or staffs, documentation; professional discussion; and administrative task/meeting by assigning specific individual during the medication error event or using supportive supervision.

## Figures and Tables

**Table 1:** Sociodemographics, work environment, and perceptual characteristics of participants.

Variables	Frequency	Percent
Gender		
Male	140	50.4
Female	138	49.6
Age		
≤25 years	34	12.2
26–30 years	190	68.3
31–40 years	43	15.5
>40 years	11	4.0
Marital status		
Single	166	59.7
Married	95	34.2
Divorced/widowed	17	6.1
Educational status		
Diploma	61	21.9
B.S.	217	78.1
Professional experience		
≤5 years	163	58.6
6–10 years	88	31.7
>10 years	27	9.7
Perceived risk of WIs		
Perceived as no/low risk	162	58.3
Perceived as high risk	116	41.7
Unit type		
Pediatric	78	28.1
Medical	73	26.3
Surgical	75	27.0
Gynecology	23	10.4
Ophthalmology	29	8.3
Day of week		
Weekday	197	70.9
Weekend	81	29.1
Shift of day		
Regular	203	73.0
Night	75	27.0
Time of medication round		
10 AM	66	23.7
12 PM	68	24.5
2 PM	69	24.8
6 PM	15	5.4
10 PM	15	5.4
12 AM	15	5.4
2 AM	15	5.4
6 AM	15	5.4

**Table 2:** Participants' frequency and percentage distribution of work interruptions during the phases of medication administration process.

Phase of MAP (in which WIs occur)	Work interruptions (by main severity category)		
	Minor	Major	Overall
Preadministration^*∗*^	371 (32.2)	394 (34.2)	765 (66.4)
Administration^*∗∗*^	45 (3.9)	69 (6.0)	114 (9.9)
Postadministration^*∗∗∗*^	157 (13.6)	116 (10.1)	273 (23.7)
Overall, *n* (%)	573 (49.7)	579 (50.3)	1152 (100.0)

^*∗*^Preparation and verification of the medication. ^*∗∗*^Delivering the medication to the patient. ^*∗∗∗*^Documentation, clarification, and in-transit medication management: between one medication and another, or between one patient's medication activity and another.

**Table 3:** The frequency and percentage distribution of the overall work interruptions by the characteristics of individuals and working environment (*n* = 1152).

Variables	Frequency	Percent
*General environmental characteristics*		
Unit type		
Pediatric	344	29.9
Medical	295	25.6
Surgical	339	29.4
Gynecology	72	6.2
Ophthalmology	102	8.9
Day of week	102	
Weekday	76.4	76.4
Weekend	23.6	23.6
Shift of day		
Regular	962	83.5
Night	190	16.5
Time of medication round		
12 PM	363	31.5
2 PM	316	27.4
6 PM	37	3.2
10 PM	31	2.7
12 AM	39	3.4
2 AM	39	3.4
6 AM	44	3.8
10 AM	283	24.6
*Individual/participant characteristics *		
Gender		
Male	577	50.1
Female	575	49.9
Age		
≤25 years	151	13.1
26–30 years	782	67.9
31–40 years	188	16.3
>40 years	31	2.7
Marital status		
Single	704	61.1
Married	381	33.1
Divorced/widowed	67	5.8
Educational status		
Diploma	206	17.9
B.S.	946	82.1
Professional experience		
≤5 years	747	64.8
6–10 years	300	26.0
>10 years	105	9.1
Perceived risk of WIs		
Perceived as no/low risk	666	57.8
Perceived as high risk	486	42.2

**Table 4:** Multivariate analysis of factors associated with major work interruptions (*n* = 278).

Variables	AOR	95% CI for AOR	*p-*value
Unit type			
Pediatric	1		
Medical	1.344	0.943–1.917	0.102
Surgical	1.854	1.328–2.588	<0.001
Others	1.293	0.863–1.938	0.213
Day of week			
Weekday	1		
Weekend	1.389	1.021–1.890	0.036
Source/initiation			
Face-to-face conversation	1		
Phone call/page	2.104	1.263–3.506	0.004
Technical	1.494	1.002–2.227	0.049
Reason/secondary task			
Direct care	1		
Indirect care	1.215	0.741–1.992	0.440
Failure resolution	2.850	1.746–4.653	<0.001
Professional discussion	2.830	1.213–6.602	0.016
Clerical	4.250	2.277–7.933	<0.001
Education/supervision	2.234	1.112–4.486	0.024
Administrative	2.255	1.167–4.355	0.015
Social/private	2.719	1.758–4.206	<0.001
Professional experience			
≤5 years	2.088	1.277–3.414	0.003
6–10 years	1.821	1.078–3.075	0.025
>10 years	1		
Perceived risk of WIs			
Perceived as no/low risk	1.979	1.504–2.605	<0.001
Perceived as high risk	1		

“Others” stands for (i) ophthalmology (special eye care both for adult and pediatric patients, neither pediatric only nor adult care only as usual) and (ii) gynecology (special reproductive organ care for female patients, neither medical only nor surgical care only as usual).
