# Corrigendum to “Work Interruption Experienced by Nurses during Medication Administration Process and Associated Factors, Northwest Ethiopia”

**DOI:** 10.1155/2019/6106932

**Published:** 2019-09-05

**Authors:** Nursing Research and Practice, Abebaw Jember Ferede, Berhanu Boru Bifftu

**Affiliations:** Department of Nursing, University of Gondar College of Medicine and Health Science, Gondar, Ethiopia

In the article titled “Work Interruption Experienced by Nurses during Medication Administration Process and Associated Factors, Northwest Ethiopia” [1], Dr. Abebaw Jember Ferede was missing from the authors' list. Dr. Ferede was the coadvisor of Dr. Getnet's thesis and contributed to designing, writing, and commenting on the study. The corrected authors' list is shown above. The correction was made at the request of the School of Nursing, but Dr. Getnet did not agree.
